# The Contribution of Executive Functions to Spelling: A study in L1 Arabic and English as a Foreign Language

**DOI:** 10.1007/s10936-026-10211-6

**Published:** 2026-02-24

**Authors:** Rana Sammour-Shehadeh, Janina Kahn-Horwitz, Anat Prior

**Affiliations:** 1 Department of Learning Disabilities, University of Haifa, Haifa, Israel; 2 Department of English Language and Literature, Oranim Academic College, Kiryat Tiv’on, Israel; 3 Department of Learning Disabilities and Edmond J. Safra Brain Research Center for Learning Disabilities, Faculty of Education, University of Haifa, Haifa, Israel

**Keywords:** Spelling, Executive functions, Foreign language, Arabic, English

## Abstract

Executive Functions (EFs) are known to play a role in academic performance, especially in reading and mathematics, but their role in spelling is not clear and has received scant research attention. The present study targets this gap in the literature and examines the contribution of EFs to spelling beyond phonological awareness (PA). It broadens the perspective by concomitantly examining spelling in L1 Arabic and in English as a foreign language. Measures of EFs (working memory, inhibition, and shifting), Arabic and English PA, and Arabic and English spelling were administered to a sample of 84 fifth graders and 80 eighth graders. Results demonstrated that working memory showed a marginally significant contribution to spelling in Arabic, but not in English, whereas inhibition and shifting did not significantly predict spelling in either language. Further, the links between EFs and spelling remained stable across the two grade levels. These findings identify working memory as a potential contributor to spelling which may vary depending on the language status, but do not support the contribution of inhibition and shifting to spelling.

## Introduction

Spelling is an essential component of literacy acquisition. Spelling words automatically and effortlessly enables writers to focus their attention on higher-level processes that are necessary for writing, such as planning and revising (Graham & Santangelo, 2014; Harris et al., 2017). On the flip side, spelling difficulties may interfere with holding the writing content in working memory, and erroneous spelling can obscure the message that writers intend to convey (Graham et al., 2002; Graham & Santangelo, 2014).

Spelling is a complex developmental skill based on various linguistic components, including phonology, morphology and orthography. Phonological awareness stands out as one of the strongest linguistic factors contributing to spelling (e.g., Al-Mannai & Everatt, 2005; Asadi et al., 2017; Bruck & Treiman, 1990; Furnes & Samuelsson, 2011; Griffith, 1991; Strattman & Hodson, 2005; Weinrich & Fay, 2007; Wolff & Gustafsson, 2022). Spelling ability also depends on cognitive skills, such as rapid automatized naming (e.g., Stainthorp et al., 2013), but currently not enough is known regarding the possible contribution of executive functions (EFs) to spelling (Czapka et al., 2019). Therefore, the main goal of the current study is to investigate whether EFs predict spelling. We examine the role of EFs in spelling in both the native language, Arabic, and a foreign language (FL), English, of elementary and middle-school children. Examining the role of EFs in spelling will broaden our theoretical perspective on spelling. Such improved understanding can have implications for spelling instruction and assessment, as the relevant cognitive factors involved can help identify children who may struggle with spelling.

## Predictors of Spelling in First and Second Language

According to the *central processing hypothesis* (Geva & Ryan, 1993; Geva & Siegel, 2000), literacy skills in first (L1) and second language (L2) rely on shared underlying cognitive and linguistic processes, such as working memory and phonological awareness. This hypothesis posits that the acquisition of such skills is not influenced by the nature of the specific orthography. The *script dependent hypothesis*, on the other hand, states that the development of literacy skills varies across languages as a function of orthographic transparency (Katz & Frost, 1992; Lallier & Carreiras, 2018). For example, Ziegler et al. (2010) found that most predictors of reading were consistent across alphabetic languages, although their importance varied depending on the transparency of the orthography (see also Gottardo et al., 2021; Rueckl et al., 2015). Geva and Siegel (2000) concluded that the central processing and the script dependent frameworks are not contradictory but rather complementary.

Spelling ability in L1 has been linked to various linguistic and cognitive factors in school-age children. One of the most influential linguistic factors is phonological awareness (PA), which refers to the awareness of the phonological units of the spoken language, including syllables, intrasyllabic units, and phonemes (Treiman, 1991). PA is strongly associated with spelling in children’s L1 in different languages, including Arabic, and across shallow and deep orthographies (Al-Mannai & Everatt, 2005; Asadi et al., 2017; Bruck & Treiman, 1990; Furnes & Samuelsson, 2011; Griffith, 1991; Moll et al., 2014; Saiegh-Haddad & Taha, 2017; Strattman & Hodson, 2005; Stuart & Masterson, 1992; Weinrich & Fay, 2007; Wolff & Gustafsson, 2022). Morphological awareness, defined as the understanding of the morphemic structure of words (Carlisle, 1995), has also been found to contribute to spelling in different languages, including Arabic and English (Angelelli et al., 2014; Asadi et al., 2017; Casalis et al., 2011; Deacon et al., 2009; Khoury-Metanis et al., 2018; Moats, 1995; Saiegh-Haddad & Taha, 2017). Orthographic knowledge, which is a multi-dimensional construct composed of word specific knowledge and general orthographic knowledge (Conrad et al., 2013; Hagiliasis et al., 2006; Rothe et al., 2014; Zarić et al., 2021), is another essential linguistic factor that plays a role in spelling (Arab-Moghaddam & Sénéchal, 2001; Asadi et al., 2017; Conrad et al., 2013; Landerl & Wimmer, 2008; Rothe et al., 2014; Zarić et al., 2021). Links between orthographic knowledge and spelling have also been found in Arabic (Asadi et al., 2017; Khoury-Metanis et al., 2018; Khoury-Metanis & Khateb, 2022).

Besides these linguistic factors, spelling seems to involve cognitive functions as well. Thus, rapid automatized naming (RAN), the ability to name a series of familiar items as quickly as possible, has been associated with spelling abilities (Furnes & Samuelsson, 2011; Savage et al., 2008; Stainthorp et al., 2013; Strattman & Hodson, 2005; Verhagen et al., 2010), but findings regarding its contribution to spelling are inconsistent (e.g., Moll et al., 2014). In Arabic, for example, Saiegh-Haddad and Taha (2017) found a connection between RAN and spelling, whereas Asadi et al. (2017) and Batnini and Uno (2015) did not. Moll et al. (2014) explored the contribution of different cognitive underpinnings (IQ, phonological short term and working memory, PA, RAN) to spelling in five orthographies with varying degrees of orthographic transparency (English, French, German, Hungarian, Finnish) and concluded that the predictive pattern was partly consistent across orthographies. Phonological processing was the best predictor of spelling in all orthographies except English, where RAN explained more variance than PA. Together, these studies imply that while there are subtle differences, the cognitive and linguistic underpinnings of spelling in different languages and orthographies are mostly shared, which aligns with the central processing hypothesis (Geva & Siegel, 2000).

Alongside the relatively large body of research on the spelling across various L1s, a small body of research has investigated the predictors of spelling in L2 (Czapka et al., 2019; Harrison et al., 2016; Jongejan et al., 2007; Marinova-Todd & Hall, 2013; Russak, 2020; Russak & Kahn-Horwitz, 2015). Jongejan et al. (2007) examined whether PA, rapid naming, working memory and syntactic awareness predict spelling in L1 and L2 English learners in Grades 1–4. They found that in the lower grades (Grades 1–2), PA and verbal working memory were significant contributors to spelling in L1 English speakers, with PA being the strongest predictor, whereas PA was the only significant predictor in L2 English learners. In the upper grades (Grades 3–4), syntactic awareness and verbal working memory contributed significantly to spelling in L1 English speakers, with syntactic awareness being the strongest predictor, whereas PA and rapid naming were significant predictors of spelling in L2 English learners, with PA being the strongest predictor. Harrison et al. (2016) investigated similar predictors in third graders and found that PA predicted word spelling for L1 English speakers, whereas both PA and rapid naming predicted word spelling for L2 learners, with PA being the strongest predictor. Czapka et al. (2019) found that PA predicted spelling in L1 German third graders, whereas both PA and lexicon size predicted spelling in L2 German learners in the same grade. The researchers also found that PA and lexicon size predicted pseudoword spelling in L1 German, whereas only PA predicted spelling in L2 German. Taken together, these studies suggest that there are commonalties as well as differences in the factors that predict L1 and L2 spelling. Importantly, PA emerges as a significant contributor to both L1 and L2 spelling.

In addition, a small number of studies examined English spelling in an FL setting and found connections between PA and spelling (Russak, 2019, 2020; Zhao et al., 2017) and between orthographic knowledge and spelling (Russak, 2020; Russak & Kahn-Horwitz, 2015; Zhao et al., 2017).

### Executive Functions and Spelling

EFs are defined as a set of high-level mental processes that are required for paying attention in order to achieve a goal and in cases when using an automatic process is inappropriate (Diamond, 2013). There are three core EFs: working memory (holding information in mind and mentally updating it), inhibition (suppressing prepotent or dominant responses), and shifting (switching between multiple tasks or mental sets; Miyake et al., 2000).

EFs are implicated in school achievement, because they support children’s ability to remember and follow complex instructions, avoid distractions, control impulsive responses, adjust when the rules change and persist at problem solving. Thus, EFs are positively linked to reading (e.g., Foy & Mann, 2013; Yeniad et al., 2012) and mathematics (e.g., Bull & Lee, 2014; Cragg & Gilmore, 2014). EFs are also expected to be important for spelling because spelling requires coordinating and shifting between several mental tasks, including holding the word to be written in mind while analyzing and sequencing its component phonemes, retrieving orthographic information from long-term memory, applying morphological rules, choosing the appropriate graphemes while rejecting the incorrect ones, and shifting between different strategies when spelling a word (Czapka et al., 2019; Lubin et al., 2016; Strattman & Hodson, 2005; Von Suchodoletz et al., 2017).

The development of executive functions has been extensively studied (Davidson et al., 2006; Diamond, 1985, 2006, 2013; Garon et al., 2008; Luna et al., 2004). Research indicates that significant improvements in inhibition occur between the ages of 3 and 7 years (Diamond, 2006), and this maturation continues through adolescence (Luna, 2004). The development of working memory also starts early, with infants as young as 9 to 12 months demonstrating an ability to update the contents of their working memory (Diamond, 1985). However, the ability to hold multiple items in mind or perform mental manipulations develops more slowly, showing a prolonged developmental trajectory (Davidson et al., 2006). Shifting, which builds on both inhibition and working memory, comes in much later in development (Davidson et al., 2006; Garon et al., 2008). Improvements in shifting, particularly in the ability to flexibly switch back and forth, are evident between the ages of 5 and 11 years, but it may only reach adult levels until 20 years of age (Cepeda et al., 2001).

Among the three core EFs, working memory (WM) has been most widely researched, but findings regarding its possible link with spelling achievements are mixed. Some studies report a positive relationship between WM and spelling in children (Asadi et al., 2017; Bourke et al., 2014; De Vita et al., 2021; Harrison et al., 2016; Jongejan et al., 2007; Khoury-Metanis & Khateb, 2024) and adults (Ormond & Cochran, 1988). For example, Asadi et al. (2017) found that WM contributed to spelling in Arabic-speaking children in first grade, but not later in elementary school. Bourke et al. (2014) reported that visuo-spatial WM contributed to spelling among English-speaking children in their first year of school, after controlling for nonverbal cognitive ability, phonological WM, visual perceptual processing and orthographic transcription skills. De Vita et al. (2021) found that Italian-speaking children in the third to fifth grade with high WM performed better than children with low WM on a real-word dictation task, but the two groups performed equally well on a non-word dictation task.

On the other hand, other studies suggest that WM might not contribute to spelling abilities (Czapka et al., 2019; Lubin et al., 2016; Strattman & Hodson, 2005; Swanson & Berninger, 1996; Vanderberg & Swanson, 2007). For example, studies investigating elementary- school children found that short term memory, but not WM, predicted handwriting and spelling (Swanson & Berninger, 1996), or that WM predicted decoding, but not spelling (Strattman & Hodson, 2005). In high-schoolers, WM contributed to higher-order writing measures but not to spelling (Vandenberg & Swanson, 2007). Thus, although a number of studies have examined the relations between WM and spelling, the results are inconclusive.

These conflicting results can be attributed to several factors. First, different age groups were included in different studies. Previous research has shown that the relationship between EFs and academic performance depends on age (Blair & Razza, 2007), and the same might be true for spelling. For example, WM may play a minimal role, if any, in skilled spellers, for whom spelling is automatic. In contrast, WM may play a greater role in novice spellers, especially when spelling unfamiliar words, as this process requires segmenting the spoken form into its constituent phonemes and processing each letter (Share, 2024).

Second, studies vary greatly in the WM tasks that were used. Whereas some studies included several WM measures (e.g., Swanson & Berninger, 1996; Vanderberg & Swanson, 2007), others included a single WM measure, either verbal (e.g., Czapka et al., 2019; Lubin et al., 2016; Ormond & Cochran, 1988) or visual-spatial (e.g., Bourke et al., 2014). Different tasks may vary in their complexity level and in the cognitive processes they tap, therefore leading to variability in the links observed between them and spelling. Third, different studies examined spelling in different languages. The relations between EFs and spelling may be modulated by language. For example, orthographic depth might modulate the relation between WM and spelling. Whereas spelling in a shallow orthography mostly requires phoneme to grapheme conversions, spelling in a deep orthography is a more complex process that requires the use and coordination of multiple sources of linguistic information and thus is expected to draw more on WM resources. Support for the different role of WM in deep compared to more transparent orthographies was found for reading skills (Malda et al., 2014), and the same might be true for spelling.

Whereas WM has been widely studied, only a limited number of studies have explored the direct contribution of inhibition and shifting to spelling. Notably, shifting, but not WM or inhibition, was found to contribute to spelling in fourth-grade French-speaking children (Lubin et al., 2016) and in third-grade German-speaking children (Czapka et al., 2019). Altemeier et al. (2008) reported that shifting was a better predictor of spelling than inhibition in elementary-school children. Finally, Von Suchodoletz et al. (2017) reported that better attention shifting was related to higher spelling achievement in German-speaking first, third and eighth graders, with some variability across the age groups in the specific spelling strategies involved. These studies suggest that shifting ability is linked to spelling accuracy, more so than inhibition. In the current study, we examine the possible contribution of all three EFs concurrently.

Importantly, most of the studies described above investigated the possible contribution of EFs to spelling within a single age group. Therefore, there is limited understanding regarding whether and how the connections between EFs and spelling change across different age groups. Von Suchodoletz et al. (2017) investigated the relations between shifting and spelling across first-, third- and eighth-grade German-speaking students. They found connections between students’ shifting and their proficiency in using the alphabetic strategy in spelling across all grade levels. However, a connection between students’ shifting and their proficiency in using the orthographic strategy was found only for third graders. Also, shifting was related to general spelling in third and eighth graders, but not in first graders. Examining a different EF, Jongejan et al. (2007) reported that WM was a significant contributor to spelling in L1 English-speaking students in Grades 1–2 as well as in Grades 3–4. Taken together, these results suggest that the connections between EFs and spelling may vary depending on the specific EF being examined, the specific spelling aspect being studied and possibly on the age group involved. The present study will compare the contribution of WM, inhibition and shifting to spelling across two different grade levels, namely fifth and eighth grades.

Additionally, most of the studies mentioned thus far examined the possible contribution of EF to spelling in children’s L1, and very little is known about these links in L2 (Czapka et al., 2019; Harrison et al., 2016; Jongejan et al., 2007). Jongejan et al. (2007) reported that WM predicted spelling in L1 English-speaking students in Grades 1 through 4 beyond other linguistic abilities, but not in L2 English learners, though WM was significantly correlated with spelling in both groups. Somewhat similarly, Harrison et al. (2016) found that WM correlated moderately with spelling in third-grade L1 English speakers and L2 English learners, but it did not predict spelling performance beyond PA and rapid naming. In a study examining WM, inhibition and shifting, Czapka et al. (2019) found that only shifting contributed to spelling real words and non-words in L1 German-speaking third graders. In contrast, for L2 German learners, inhibition contributed to spelling words and shifting to spelling non-words. After the addition of language-related skills to the model, the contribution of shifting disappeared in both groups, whereas inhibition remained a significant predictor for L2 German learners. The present study will compare the contribution of WM, inhibition and shifting to spelling in L1 Arabic and English as an FL, whereas the previous studies have examined L2 spelling in immersion settings. Spelling in an FL setting is expected to be more demanding than spelling in an L1 setting, due to the limited amount of exposure to the language and because it necessitates managing interference from L1, and thus it may require a greater engagement of EFs.

## The Current Study

The current study addresses two research questions that to date have not received adequate attention in the literature. The first research question is: Do EFs contribute to spelling beyond PA in both the L1, Arabic, and in English as an FL? If so, which of the components (WM, inhibition, and shifting) makes a significant contribution? One important advantage of the current design is that we concurrently investigate the possible influence of all three EFs—WM, inhibition, and shifting—on spelling in a Semitic language (Arabic) that uses an abjad writing system and on spelling in a foreign language (English). Identifying similar predictors of spelling across the two languages would lend support to *the central processing hypothesis*, whereas identifying different predictors would support *the script dependent hypothesis*.

We chose to incorporate PA as a linguistic predictor in the current study because it has been identified as a robust contributor to spelling in most previous research (e.g., Al-Mannai & Everatt, 2005; Furnes & Samuelsson, 2011; Moll et al., 2014). Further, it is relatively simple to construct comparable measures of PA across the two languages investigated in the current study (in contrast to measures of morphological awareness or orthographic knowledge).

The second research question we address is: Do the relations between EFs and spelling vary across the two grade levels? The study can provide insights into how different EF components may impact academic skills and their varying influence at different stages of schooling. For example, younger children in fifth grade might rely more on EFs that develop relatively early, such as WM or inhibition, whereas older children in eighth grade might depend more on shifting, which develops later than the other two.

## Method

### Participants

A total of 164 native Arabic-speaking students participated in the study: 84 fifth graders with a mean age of 10 years and 9 months (38 girls) and 80 eighth graders with a mean age of 13 years and 8 months (51 girls). The sample consisted of students with varying ability levels, including those diagnosed with a learning disability, to reflect the natural variation present in the classroom settings from which the sample was drawn. Students participated on a voluntary basis. Only those whose parents signed a consent form participated. Students were excluded from the study if one of their parents was a native speaker of a language other than Arabic. They were sampled from two elementary schools and one middle school, situated in the north of Israel. The sample comprised students from four fifth-grade classes and four eighth-grade classes across the three schools. The schools were characterized by low socioeconomic background.

The participants study Hebrew as an L2 and English as an FL. In Israel, English literacy instruction usually starts in the third grade after exposing the students to the oral language for one year or more. English spelling instruction initially includes teaching letter names and sound-letter matching for most of the single letter graphemes and some frequent multi-letter graphemes. After that, students are required to memorize the spelling of words for dictation tests (Russak & Kahn-Horwitz, 2015). Students receive an average of 3–4 h of English instruction per week in elementary and middle school (Israel Ministry of Education, 2022).

### Measures

#### Linguistic Measures

**PA in Arabic** (Asadi, 2012). PA was assessed using a Phoneme deletion task. Participants were orally presented with a word, instructed to repeat it, and then delete a single phoneme from the beginning, middle or end of the word. The test consists of 18 mono- and disyllabic words, most of which are shared across Spoken Arabic and Modern Standard Arabic, and the rest taken from Spoken Arabic. The number of correct answers was used as a measure of PA. The reliability of the test (*Cronbach’s alpha*) is 0.94 and 0.91 for fifth and eighth grade, respectively. All reliability measures reported throughout the manuscript were based on the present sample.

**PA in English**. This was examined using the Elision subtest of the Comprehensive Test of Phonological Processing (CTOPP; Wagner et al., 1999). Participants were orally presented with a word, instructed to repeat it and then remove a single phoneme from the beginning, middle or end of the word. The test includes 20 items. The number of correct answers was used as a measure of PA. The reliability of the test (*Cronbach’s alpha*) is 0.88 for the fifth grade and 0.92 for the eighth grade.

#### Executive Functions

**Inhibition**. The Spatial Stroop task *(*also called Simon Arrows), adopted from Bialystok et al. (2008), was used to assess inhibition. In each trial, an arrow pointing either left or right appears on the screen. In the basic condition, consisting of 24 trials, the arrow appears in the center of the screen, and participants are instructed to respond by button press to the direction of the arrow as quickly and accurately as possible. In the conflict condition, the target arrows are presented on the left or right sides of the display, creating congruent trials when the direction and position correspond, and incongruent trials when they are in conflict. The conflict condition consists of 72 trials, with equal numbers of congruent and incongruent trials. Participants were instructed to press the button indicating the direction of the arrow irrespective of the position. Instructions in each of the conditions are followed by four practice trials in which feedback was given. In addition, when participants responded incorrectly throughout the task, a short buzzer sound was played. Inhibitory abilities were indicated by differences in performance between congruent and incongruent trials in the conflict block, using the bin scoring method (Hughes et al., 2014). The bin score measure represents both RT and accuracy for costs in performance when comparing the challenging versus the easier task condition. Each incongruent trial RT (“difficult” trial) was subtracted from the participant’s average RT for the congruent trials (“easy” trials). These difference scores across all participants in the group were rank-ordered and distributed in bins (ranging in value from 1 to 10). Each participant received a “bin score”, which is the sum of the number of his or her trials placed in each bin. Error trials received a score of 20 and were added to each participant’s bin score. Thus, the bin score incorporates costs to performance across both RT and accuracy. Higher bin scores indicate that a participant’s performance suffered to a greater extent by the added cognitive burden in the incongruent condition.

**Working memory**. The Color span backwards (Hasselhorn et al., 2012) was used to assess the central-executive WM. In this computerized task, participants are required to memorize a sequence of visually presented circles with different colors and recall them in the reverse order, by pressing keys corresponding to each color. The task begins with sequences including two colors and proceeds to a maximal sequence length of eight. If participants correctly reproduce two sequences out of three of the same length, they proceed to the next sequence length; otherwise, the task is terminated. After the instructions, participants are given two practice trials with feedback to make sure they understand the task requirements. Participants’ WM score is the number of correct responses throughout the task.

**Shifting**. A computerized version of the Dimensional Change Card Sort (DCCS; Zelazo, 2006) based on the NIH (National Institute of Health) toolbox were used to measure attention shifting. Stimuli are presented on a computer screen. Two receptacles, one marked with a blue circle, and one marked by a red square, serve as the sorting bins and are presented in the bottom right and left corners of the screen, respectively, throughout the task. Trials begin with a presented cue that indicates the sorting rule for that trial. The cue for a color trial is composed of a series of blue and red shapes whereas the cue for a shape trial is composed of a series of gray squares and circles. Cues are followed by centrally presented test stimuli. Test stimuli are presented for 10 s or until a response is given, and they include red circles and blue squares, such that each stimulus matches each target on a single dimension (color or shape). The task begins with four practice trials on which participants receive feedback. In the first experimental pre-switch block, which includes five trials, participants are asked to sort the stimuli by color. In the second experimental post-switch block, which includes five trials, participants are required to sort the stimuli by shape. The final block is a mixed block, in which participants are instructed to sort the stimuli according to the cue appearing on each trial. This block includes 23 shape trials and seven color trials. Accuracy rates and reaction time were recorded, and scores were computed according to the NIH Toolbox Technical Manual (Slotkin et al., 2012). Participants receive a single score that incorporates both accuracy and RT.

#### Spelling

**Spelling in Arabic** (Asadi et al., 2017). Arabic spelling was assessed using a vowelized word test that consists of 24 items, with the words varying in length from one to four syllables. The words were dictated to the students who were instructed to write them as accurately as possible. The task was scored by summing the number of correctly spelled words. The students received 1 point if the spelled word was composed of correct letters, even if short vowels were not represented. The reliability of the test (*Cronbach’s alpha*) is 0.86 for the fifth grade and 0.78 for the eighth grade.

**Spelling in English**. The Test of Written Spelling, Fifth Edition–Form A (TWS-5; Larsen et al., 2013) was administered to the participants. The original task consists of 50 words that vary in length, drawn from eight basal spelling series and graded word lists, encompassing both regular and irregular words. The students were asked to write the words as accurately as possible. Since most Arab students are not familiar with some of the words that appear in the list, based on previous data collected by Kahn-Horwitz (2020), only the first twenty words were used with fifth graders and the first thirty with eighth graders. The number of correctly spelled words was used as a measure of task performance. The words were dictated by the first author, a native Arabic speaker, with each word read twice. The reliability of the test (*Cronbach’s alpha*) is 0.83 for the fifth grade and 0.91 for the eighth grade.

##### Procedure

Testing took place in the second term of the school year, after obtaining Ethics approval from the Israeli Ministry of Education Chief Scientist’s office, the University of Haifa’s institutional review board, and informed consent from the students’ parents. The Arabic and English spelling tests were administered in a group setting during two separate sessions, with testing conducted in whole classes comprising 20 to 26 students. PA and EF tasks were individually administered to participants in a quiet room at school over one session. Testing was conducted by the first author and another examiner who underwent special training for the purpose of the study.^1^

## Results

Table 1 presents performance on the study variables by grade. As a preliminary step, we first directly compared performance across the two grade levels using t tests.

**Table 1:** Mean scores, standard deviations, minimum and maximum values on predictor variables and spelling measures

	Fifth graders		Eighth graders		
	M (SD)	Min-Max	M (SD)	Min-Max	t
Arabic PA	10.45 (5.99)	1–18	12.05 (5.06)	2–18	1.85~
English PA	14.94 (4.49)	4–20	16.33 (4.59)	4–20	1.95~
WM	5.32 (1.92)	0–10	6.03 (1.99)	0–14	2.3*
Inhibition	251.19 (49.18)	145–400	247 (47.39)	161–369	0.55
Shifting	5.45 (1.71)	2.25–10.5	6.39 (1.72)	2–8.9.9	3.46***
Arabic spelling	13.77 (5.11)	2–24	14.58 (4.62)	4–23	1.03
English spelling	9.27 (3.93)	0–16	12.8 (6.25)	3–20	4.3***

~*p* <.07. *p <.05. **p <.01. ***p <.001

Across all predictor tasks, performance was numerically higher in eighth grade compared to fifth grade, likely due to maturation and schooling, with most effects being significant or approaching significance except for two effects. In addition, PA was higher in English, the foreign language, than in Arabic, the L1 (*t*(163) = −11.02, *p* <.001), which seems surprising. We ascribe this pattern to the difference in writing systems, and specifically to the fact that Arabic is an abjad, in which vowels are only sporadically represented in the written language (Abu-Rabia & Siegel, 2003). A similar pattern was found in the PA of a comparable sample of native speakers of Hebrew, also an abjad, in their L1 and in English, studied as an FL (Prior et al., 2020).

As a next step, before running the LME analysis, we calculated Pearson correlations for the following variables: Arabic PA, English PA, EFs (WM, inhibition, and shifting) and spelling in Arabic and English (see Table 2).

**Table 2:** Correlations between Predictor Variables and Spelling Measures for the Fifth Graders (above diagonal) and the Eighth Graders (below diagonal)

1. Arabic PA	1	2	3	4	5	6	7
		0.53^**^	0.27^*^	− 0.09	0.25^*^	0.53^**^	0.53^**^
2. English PA	0.47^**^		0.40^**^	− 0.17	0.26^*^	0.47^**^	0.48^**^
3. WM	0.43^**^	0.27^*^		− 0.07	0.28^*^	0.36^**^	0.36^**^
4. Inhibition	− 0.32^**^	− 0.08	− 0.20		− 0.17	− 0.16	− 0.20
5. Shifting	0.11	0.08	0.23^*^	− 0.07		0.22	0.22
6. Arabic spelling	0.38^**^	0.42^**^	0.35^**^	− 0.24^*^	0.12		0.73^**^
7. English spelling	0.32^**^	0.50^**^	0.11	− 0.01	− 0.04	0.47^**^	

*p <.05. **p <.01

### Analysis Approach

Spelling accuracy was analyzed using linear mixed effects (LME) model (Baayen et al., 2008) in R (R Core Team, 2018), with the lme4 library (Version 3.5.1), using the binomial distribution. LME models are preferred in the current design over Analyses of Variance, because they retain full information of performance, without the need for averaging across items or participants (Baayen et al., 2008).

The model included random intercepts for subject and item, as models with more complex random structures failed to converge. Arabic PA (continuous) and English PA (continuous) were entered as fixed factors, to control for participants’ linguistic abilities. Language (Arabic, English; a categorical factor with Arabic set as the reference); grade (Grade 5, Grade 8; an ordinal factor with Grade 5 set as the reference); and EFs - WM, inhibition and shifting (continuous) were entered as the fixed factors of interest. All continuous variables were centered to allow for straightforward interpretation of effects. The model also included the two-way interactions of Language with EFs (WM, inhibition and shifting) to address the first research question, namely whether EFs are differentially recruited to support spelling in the native vs. a foreign language. The model also included the two-way interactions of grade with EFs, to address the second research question, namely whether EFs were differentially recruited to support spelling by younger and older children. Table 3 presents the LME model.

**Table 3:** LME Model Predicting Spelling

Fixed effects	b	SE	z	*P*
(intercept)	0.53	0.43	1.24	0.21
Arabic PA	0.36	0.1	3.5	< 0.001***
English PA	0.43	0.1	4.27	< 0.001***
Grade (8)	−0.03	0.19	−0.18	0.86
Language (English)	−1.67	0.55	−3.03	0.002**
Working Memory	0.29	0.14	2.11	0.03*
Inhibition	−0.19	0.12	−1.56	0.12
Shifting	0.04	0.13	0.31	0.76
Language (Eng) * Grade (8)	0.55	0.13	4.05	< 0.001***
Language (Eng) * WM	−0.12	0.07	−1.78	0.07~
Language (Eng) * Inhibition	0.11	0.06	1.7	0.09~
Language (Eng) * Shifting	−0.08	0.07	−1.17	0.24
Grade (8) * Working memory	−0.13	0.18	−0.71	0.48
Grade (8) * Inhibition	0.13	0.17	0.75	0.45
Grade (8) * Shifting	−0.06	0.18	−0.32	0.75
Random effects	Variance		SD	
Item (intercept)	3.9		1.98	
Subject (intercept)	0.94		0.97	

*Note. b*= effect sizes; *SE*= standard errors; *z* = z values

~*p* <.09. *p <.05. **p <.01. ***p <.001

*P*-values for all fixed main effects and interactions were determined using the *anova* function from the ‘stats’ package, which calculates a Type III ANOVA table with Satterthwaite’s method (Table 4).

**Table 4:** ANOVA for Accuracy

Arabic PA	F	*P*
	12.24	< 0.001***
English PA	18.22	< 0.001***
Grade	1.53	0.22
Language	6.16	0.01*
Working Memory	2.85	0.09~
Inhibition	0.75	0.39
Shifting	0.08	0.78
Language * Grade	16.42	< 0.001***
Language * WM	3.18	0.07~
Language * Inhibition	2.88	0.09~
Language * Shifting	1.37	0.24
Grade * Working memory	0.5	0.48
Grade * Inhibition	0.57	0.45
Grade * Shifting	0.1	0.75

~*p* <.09, *p <.05, **p <.01, ***p <.001

The model yielded a significant main effect of language, as children were more accurate in Arabic than in English spelling. As expected, PA in both Arabic and English contributed significantly to spelling accuracy. Examining the contribution of EFs to spelling revealed that WM made a marginally significant contribution, whereas inhibition and shifting did not contribute significantly. Further, the interaction between Grade and Language was significant, and the interactions between WM and Language and between inhibition and language were marginally significant. No other interactions were significant.

**Fig. 1:**
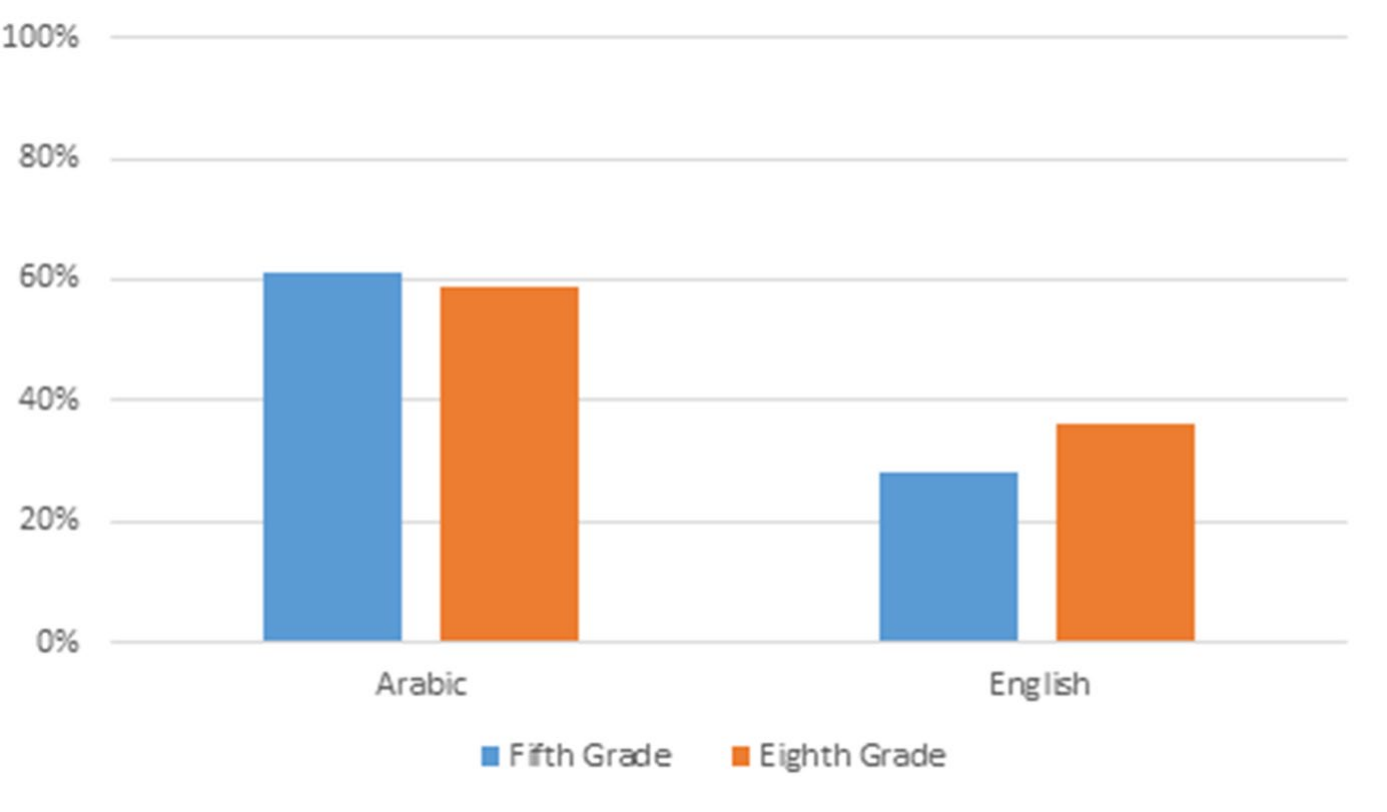
Estimated Means (SEM) for Spelling Accuracy, by Language and Grade

We next probed the significant two-way interaction between language and grade, using the *testInteractions* function, with Bonferroni adjustment. This revealed that whereas the spelling accuracy of fifth and eighth graders did not differ in Arabic (χ^*2*^(1) = 0.3, *p =* 1), eighth graders were more accurate than fifth graders in English spelling (χ^2^(1) = 7.21, *p*<.05, see Fig. 1).

We also probed the marginally significant two-way interaction between WM and language due to the theoretical interest. Results showed that WM made a marginally significant contribution to spelling in Arabic (χ^*2*^(1) = 4.89, *p =*.054~), but not in English (χ^*2*^(1) = 0.99, *p =*.64). Probing the marginally significant two-way interaction between inhibition and language revealed that inhibition did not contribute to spelling in either language, although the slope of the effect was numerically larger in Arabic than in English.

## Discussion

The present study examined the contribution of EFs to spelling beyond PA in fifth and eighth grades. Spelling was tested in Arabic, the students’ L1, as well as in English, which they study as an FL. As expected, Arabic spelling was more accurate than English spelling. Further, the fifth and eighth graders performed equally well on Arabic spelling, but the older children had better English spelling than younger children. Consistent with previous research, PA in both Arabic and English was a significant contributor to spelling, with better PA predicting higher spelling accuracy.

### The Contribution of EFs to Spelling across Languages

Our study found a marginally significant contribution of WM to spelling performance. While the p-value did not reach conventional levels of significance, the trend suggests that there may be a relationship between WM and spelling abilities. As explained above, spelling requires the simultaneous processing, storage and retrieval of information, and consequently it places a demand on WM. The marginally significant contribution of WM to spelling may be partly explained by the contribution of PA, which was used as a control variable. PA was found to be a robust predictor of spelling and was also correlated with WM. This relationship between PA and WM may have attenuated the apparent contribution of WM to spelling in our model.

In addition, we found a marginally significant interaction between WM and language, indicating that WM made a marginally significant contribution to spelling in Arabic, the L1, but not in English, the FL. Thus, it seems that WM is not a consistent predictor across the two languages. This finding challenges the *central processing hypothesis* (Geva & Ryan, 1993; Geva & Siegel, 2000), which posits that literacy skills in L1 and L2 are based on shared cognitive and linguistic processes. Conversely, it lends more support to the *script dependent hypothesis*, which argues that the development of literacy skills varies across orthographies, depending on their orthographic depth, as well as educational settings (Gottardo et al., 2021; Katz & Frost, 1992; Lallier & Carreiras, 2018).

Why might WM predict spelling in Arabic but not in English? One notable difference between the two languages relates to the linguistic proficiency of students, who demonstrate higher proficiency in Arabic, their L1, compared to English. Thus, it is possible that when participants spell in their L1, they rely on multiple sources of linguistic information (Saeigh-Haddad et al., 2023), which they simultaneously process, store and retrieve, thus drawing on WM resources. We tentatively suggest that when spelling in a foreign language, especially one in which they are at a low level of proficiency, learners may have fewer sources of information (due to the limited exposure to the language) and therefore may rely on one strategy only. In the case of English spelling, participants may depend largely on rote memorization, which may reflect the instructional approach typically used in their context. In this approach students are first taught letters and common digraphs, followed by the memorization of word spellings for dictation tasks (Russak & Kahn-Horwitz, 2015). This less sophisticated approach to spelling might require children to rely to a lesser degree on their working memory resources. However, further research is necessary to investigate this possibility. Czapka et al. (2019), who found that EFs explained a small amount of variance in spelling compared to linguistic factors, suggested that language skills are initially the primary factor influencing spelling, and their significant impact persists until developing language processing skills attain a certain level of proficiency, at which point cognitive resources could be reallocated to EFs.

Another notable distinction between the two languages is their orthographic depth, with English having a deep orthography and Arabic having a relatively shallow one. Given that spelling in a shallow orthography mostly involves converting phonemes to graphemes whereas spelling in a deep orthography is a more complex process requiring the use and coordination of various sources of linguistic information, one would expect WM to play a larger role in English than in Arabic. However, the opposite was found. As noted above, it may be that the participants’ proficiency level in English was not high enough for spelling to draw on multiple linguistic sources. As a result, the spelling process may have involved fewer strategic or integrative demands, thereby reducing the involvement of working memory. Students may have relied on rote memorization, reflecting the method of spelling instruction typically used in their context (Russak & Kahn-Horwitz, 2015), which may reduce the cognitive demands on working memory. These findings underscore the need for future research to consider the role of language characteristics, such as orthographic depth, in understanding the cognitive processes underlying spelling.

The study also revealed that inhibition did not contribute to spelling in either language. This finding is consistent with previous studies which did not identify a unique contribution of inhibition to spelling (Altemeier et al., 2008; Czapka et al., 2019; Lubin et al., 2016). Further, shifting did not predict spelling for either language, in line with some previous research (Czapka et al., 2019; Soto et al., 2021), and in contradiction of others (Lubin et al., 2106; Von Suchodoletz et al., 2017). As described above for WM, variability in shifting tasks and participant characteristic might explain these different patterns across studies (as proposed by Yeniad et al., 2013, for the links between shifting and academic achievements more generally). In addition, because of the shared variance across the constructs of inhibition and shifting (Miyake et al., 2000), the specific tasks chosen for each construct across different studies (Altemeier et al., 2008; Czapka et al., 2019; Lubin et al., 2016) might influence the outcome of how statistical models assign variance. Thus, in the current study both inhibition and shifting measures were computerized and were scored based on both RT and accuracy, leading to similar distributions. In contrast, previous studies used a mix of computerized and paper and pencil tasks across inhibition and shifting, which could lead to different outcome patterns. Clearly, this is a question ripe for future research. Thus, for inhibition and shifting, similar patterns are observed across Arabic and English, with neither variable significantly contributing to spelling in either language. This finding supports the *central processing hypothesis*.

### The Contribution of EFs to Spelling across Grades

In our study, the interactions between each of the three EFs and grade were not significant, meaning that the relationship between EFs and spelling did not vary significantly between fifth and eighth grade. This finding suggests that the link between EFs and spelling is relatively stable and does not change substantially during this developmental period. However, we might get different results depending on the specific task used. For example, it has been demonstrated that different complex inhibition tasks show different ages of mastery, suggesting potential variations in cognitive demands (Best & Miller, 2010). Further, we might obtain different findings if we examine a broader age range, beginning from early/middle childhood. Younger children typically undergo rapid cognitive development, including in EFs (Best & Miller, 2010; Garon et al., 2008), which could have a more pronounced impact on spelling.

As noted earlier, only a limited number of studies have explored the links between EFs and spelling across different age groups. Our results align with those of Jongejan et al. (2007), showing consistent relationships between WM and spelling across diverse grade levels, despite differences in the specific grade levels examined in each study. On the other hand, our findings contradict those of Von Suchodoletz et al. (2017). While Von Suchodoletz et al. (2017) demonstrated some variability in the relationship between shifting and spelling across different grades, our study revealed a stable pattern in which shifting was not associated with spelling across the two grades. Future research could further explore the developmental trajectory of EFs and their contribution to spelling across different grade levels.

### Limitations and Future Research

The current study extends our understanding of the underlying skills contributing to spelling accuracy by examining spelling in two languages and including measures of PA and all three EFs in the same design, across two grade levels. However, several limitations are worth mentioning. First, although we focused on the contribution of EFs to spelling, a single measure was used for each EF component and for spelling. Second, the study did not include additional linguistic predictors of spelling, apart from PA, such as morphological awareness and orthographic knowledge. Another limitation is the exclusion of Hebrew from the analysis, despite it being part of the students’ linguistic repertoire. Finally, due to the cross-sectional design of our study, we did not follow students over time and thus could not examine whether the links between EFs and spelling change during the different developmental stages.

To further promote our understanding of the relations between EFs and spelling, future research should include more than one measure for each EF component. Each EF component may manifest in different ways depending on the task demands, cognitive processes involved, and the specific context. Therefore, employing multiple measures allows for a more accurate assessment of EF components, ensures that their various dimensions are adequately captured, and enhances the validity of the findings.

Future research should also explore the relative contributions of additional linguistic predictors, such as morphological awareness and orthographic knowledge, alongside PA, to spelling performance. Incorporating these factors would offer a more comprehensive understanding of how various linguistic skills interact to influence spelling. Including Hebrew in the analysis may further enrich the findings, given that participants study it as an L2, and it forms part of their broader language development.

In addition, future studies focusing specifically on the links between WM and spelling could track the role of WM longitudinally among both L1 and EFL spellers. This would help establish whether the contribution of WM diminishes as spelling becomes more automatic.

Finally, cross-linguistic comparisons should be considered. Specifically, examining other language pairs with varying levels of orthographic depth could help determine whether the patterns of commonalities and differences observed between Arabic and English hold across languages. Such investigations would contribute to a broader and more nuanced understanding of the cognitive and linguistic predictors of spelling.

## Conclusions

In the current study, PA emerged as a robust predictor of spelling in both Arabic and English. The contribution of WM to spelling beyond PA was marginally significant, possibly due to the significant correlations between PA and WM, which may have attenuated the apparent contribution of WM to spelling. Further, WM made a marginally significant contribution to spelling in Arabic but not in English, highlighting the need to consider linguistic characteristics when studying cognitive factors in spelling. Thus, we identified both commonalities and divergences in the predictors of spelling between Arabic and English. This finding supports the existence of shared mechanisms across languages (Geva & Ryan, 1993) as well as processes specific to each orthography (Gottardo et al., 2021; Ziegler et al., 2010). Language proficiency might be one of the factors modulating the predictors of spelling, making it a crucial factor to consider when examining spelling beyond L1. The stability of the relationship between EFs and spelling across fifth and eighth grades suggests a consistent link during this developmental period.

These findings have several practical implications. First, in terms of spelling assessment, educators and practitioners aiming to understand learners’ spelling difficulties might consider evaluating their WM, as it could help identify possible cognitive underpinnings of the observed performance. By evaluating WM, educators can differentiate between spelling challenges rooted in cognitive versus linguistic deficits, allowing for more targeted support. In addition, early assessment of WM, alongside other linguistic factors such as PA, might enable practitioners to identify students who are at risk for developing spelling difficulties more effectively. Early identification of deficits in these areas can guide timely and targeted interventions, potentially mitigating future challenges. Finally, in light of the current controversy around the efficacy and generality of WM training (Melby-Lervag & Hulme, 2016; Novick et al., 2019), it is difficult at this point to recommend specific interventions. However, literacy practitioners should offer learners WM scaffolding to support spelling development and incorporate evidence-based training protocols if and when they are identified.

## Data Availability

The data that support the findings of this study are openly available in the Open Science Framework (OSF) at https://osf.io/c5b4m/?view_only=e3ca983e0560481683670d9df10c2bf7.

## Footnotes

1 Students also completed word-reading tasks in Arabic and Hebrew and orthographic knowledge, vocabulary, word identification and pseudoword spelling tasks in English. These tasks were not analyzed in the present study.
